# Grown up congenital hearts

**DOI:** 10.5935/1678-9741.20150041

**Published:** 2015

**Authors:** Jane Sommerville

**Affiliations:** 1 Emeritus Professor of Cardiology, Imperial College, London, England.

The exploits and successes of cardiac surgery for congenital heart defects over six decades has created a new population of cardiac patients - the Grown Up Congenital Hearts (GUCHs) which has many names in different countries. From successful cardiac surgery comes the refinement of other specialties - paediatric cardiology, anaesthesia, intensive care, sophisticated imaging, who looks after the GUCHs, and where are the optimal medical services for the adults (adolescents) with congenital heart diseases. At least 90% of children born with such defects can be expected to reach adulthood in regions/countries or areas where there has been established good paediatric cardiac surgery. Indeed most lesions now can be treated with success and survival. The notion of "total correction" should be dispelled as a dream of surgeons for many years. There are conditions such as closure of the persistent duct, certain ventricular septal defects and a few other simple defects which can be considered as cured of the need for future medical care. Most long-term survivors with congenital heart disease, particularly the complex, need expert medical care for life. Thus their needs must be recognised with provision of funded and staffed specialised units with necessary expertise, taking on the role of education of the problem in the region. Not every region needs a unit but with support services, research and provision of the various levels of needs of the patients.

Numbers of units depend on the size and geographical features of the country or area as well as the size of the GUCH population. Too many units lead to loss of expertise and education by diluting numbers. No one in any country knows the true numbers of GUCH patients. Predictions are not true anywhere. John Keith in the late 1950s recognised the need to have a service as his children survived, and so sent John Evans to the Toronto General Hospital to establish such a service, separate from paediatric cardiology in the Children's Hospital. So wise. In actual fact not much was established and the concerned doctor left to be a business magnate. 

Joe Perloff, influenced deeply by Paul Wood in his London training, fascinated by congenital heart disease, recognised GUCH needs in Los Angeles, established with difficult a service as did the National Heart Hospital in London with the opening of an adolescent cardiac unit in 1975 receiving the patients from Dr Richard Bonham-Carter, operated by Dr David Waterston in Hospital for Sick Children (Great Ormond Street). Services grew into Grown Up Congenital Heart unit - called after Paul Wood and rebuilt following the destruction when the Heart Hospital merged into the Brompton Hospital.

READ ARTICLE ON PAGE 373

Establishing such a unit has always been difficult for different reasons in different countries and remains so. It was facilitated by having a funded Health Service. It was easy in Malta because the principal doctors wanted it. It remains difficult in the USA where provision is only in a few isolated units. Recognition even that there is this sub specialty of cardiology has been nearly impossible in many countries; even when they have good paediatric cardiac services or because they have, has helped GUCHs since the paediatric cardiologists frequently reserve a parental right to continue looking after their patients into adulthood. It cannot be imagined that paediatricians would consider that an adult trained consultant could have a right to care for an infant. Most would not wish to do so in any specialty, so why should the paediatricians consider their ability (right) to care for adults, because they know the anatomy. In Canada, Japan and a number of European countries including the United Kingdom, it has been recognised that there needs to be a national service controlled and funded for the specialty of Grown Ups with congenital heart abnormalities as part of adult cardiology which is useful as many will acquire the cardiovascular diseases of the population.

